# When ignorance is bliss: weight perception, body mass index and quality of life in adolescents

**DOI:** 10.1038/ijo.2014.78

**Published:** 2014-06-17

**Authors:** J Hayward, L Millar, S Petersen, B Swinburn, A J Lewis

**Affiliations:** 1 WHO Collaborating Centre for Obesity Prevention, Faculty of Health, Deakin University, Geelong, Victoria, Australia; 2 Child and Adolescent Psychiatry, Clinical Sciences, Umeå University, Umeå, Sweden; 3 School of Population Health, University of Auckland, Auckland, New Zealand; 4 School of Psychology, Faculty of Health, Deakin University, Geelong, Victoria, Australia

## Abstract

**Background/Objectives:**

Body weight is negatively associated with adolescent Health-Related Quality of Life (HRQoL). Despite this well-established relationship, some adolescents with obesity do not display the expected HRQoL decreases. This study hypothesised weight perception as a moderator of the association between weight status and adolescent HRQoL.

**Subjects/Methods:**

Subjects were secondary school students from an obesity prevention project in the Barwon South-West region of Victoria, Australia, entitled *It's Your Move* (*N=*3040). Measures included standardised body mass index (*BMI-z*; World Health Organization growth standards), weight perception and HRQoL, measured by the Paediatric Quality of Life Inventory. Linear regression and average marginal effect analyses were conducted on cross-sectional baseline data to determine the significance of any interaction between weight perception and measured weight status in shaping adolescent HRQoL.

**Results:**

The *BMI-z*/perceived weight status interaction was significantly associated with adolescent HRQoL outcomes. Adolescents with *BMI z*-scores in the overweight/obesity range who perceived themselves as overweight had lower HRQoL than those who perceived themselves as ‘about right.' Conversely, adolescents with BMI scores in the lower end of the normal range or in the thinness range who perceived themselves as underweight had lower HRQoL than those with ‘about right' perceptions.

**Conclusions:**

This was the first study to report third-variable impacts of a body-perception variable on the relationship between adolescent weight status and HRQoL. Adolescents' weight perceptions significantly moderated the relationship between overweight/obesity and reduced HRQoL. Adolescents who were outside the normal weight range and misperceived their objectively measured weight status enjoyed a higher HRQoL than adolescents whose weight perception was concordant with their actual weight status. These findings suggest that practitioners may need to exercise caution when educating adolescents about their weight status, as such ‘reality checks' may negatively impact on adolescent HRQoL. It is suggested that more research be conducted to examine this potential effect.

## Introduction

The negative impact of obesity on the physical and psychiatric health and well-being of individuals has been well documented.^1,2^ Adolescent obesity is a chronic condition and a known risk factor for the later development of other diseases.^3^ Recently, studies have broadened the focus to include physical as well as psychological outcomes associated with obesity in adolescence. These studies consistently identify negative associations between weight status and subjective measures of functioning and well-being.^4^

The 2011 Youth Risk Behaviour Surveillance survey from the United States reported high rates of overweight and obesity among high-school students. The survey found 15.2% of students to be obese and 13% to be overweight.^5^ Similar prevalence figures have been recorded among Australian adolescents; combined overweight/obesity prevalence was 28% in 2008.^6^ Obesity during this developmental period is associated with increases in blood pressure, high cholesterol levels, metabolic risk, cardiovascular disease and asthma.^1^

Recently, studies have gone beyond the medical sequelae to investigate outcomes of perceived functional health and well-being, or Health-Related Quality of Life (HRQoL), associated with adolescent obesity.^4^ HRQoL is a subjective experience influenced by the individual's unique perspective on their own lifestyle, hopes, dreams and past experiences.^7^ Generally, HRQoL covers at least the following three core domains physical, emotional and social functioning, and well-being.^8^

There is a well-established negative association between body weight and HRQoL in adolescents.^4,9,10^ In rare instances, studies have failed to reproduce this association; however, these studies have either been underpowered,^11,12^ have used unconventional measures^13^ or have analysed data from samples with highly restricted body mass index (BMI) ranges.^11^

Despite the apparent strength and consistency of the association between adolescent obesity and HRQoL, some studies have found that some adolescents with obesity do not experience an associated decrease in HRQoL, suggesting that there may be significant moderators of this relationship.^14^ Identifying individual differences that may protect adolescents from obesity-related reductions in HRQoL could aide in the development of targeted intervention and in the optimal public health approach to obesity in adolescents.^15^

In this study, we focus on weight perception as a potential moderator of the relationship between overweight/obesity and HRQoL. Weight perception is defined as the subjective appraisal of actual weight status, and is impacted by social and ethnic factors.^16^ Weight perceptions may be conceptualised as a person's perception of whether they are heavier than (overweight perception), lighter than (underweight perception) or about equal to (‘right weight' perception) the right body weight.^17^

Children with objectively high BMI commonly describe their own weight inaccurately and avoid using labels that reflect extreme obesity.^18^ These individuals are found to often misperceive their weight as ‘about right', which may serve to protect the adolescent from negative stigma. Cross-cultural studies have explored the global expansion of weight-related stigma, finding that ‘fat-stigmatising' beliefs not only persist among western societies but also have spread to other cultures that were previously considered to hold ‘fat-positive' beliefs.^19^ People often describe individuals with obesity as personal and social failures, and the victims of disease. If an adolescent presents with an extreme weight status (obesity or extreme thinness), they are likely to be subject to these stigma in everyday life.^20^ Accurate perception of these weight categories, combined with identification with these stigmatised, non-ideal body types, may lead to internalisation of the negative thoughts surrounding abnormal body weight and an associated low HRQoL. HRQoL may therefore be highest where adolescents misperceive their objective extreme weight statuses, and lowest where they accurately perceive it. Previous studies have shown similar effects in Canadian adults, with objectively overweight participants who perceived themselves as overweight had poorer self-rated health and life satisfaction,^21^ and in Mexican University students, where participants who reported perceptions of overweight had comparatively low HRQoL.^22^

Accordingly, this study examines whether weight perception moderates the association between *BMI-z*, and HRQoL. It was hypothesised that:

1. Adolescents with high *BMI-z* who accurately perceive their weight status as overweight/very overweight would report lower HRQoL than adolescents with high *BMI-z* who inaccurately perceive their weight status as normal.

2. Adolescents with low *BMI-z* who accurately perceive their weight status as underweight/very underweight would report lower HRQoL than adolescents with low *BMI-z* who inaccurately perceive their weight status as normal.

## Materials and methods

### Participants

The data analysed in this study were collected during the baseline phase of *It's Your Move* (IYM), a large-sample, school-based, obesity prevention intervention programme conducted in 12 secondary schools from the Barwon South-West region of Victoria, Australia. IYM aimed to prevent childhood obesity by building the capacity of the community to promote healthy eating and physical activity. Data were collected from *N*=3040 students (original sample *N*=5961, response rate 51%) with a mean age of 14.6 years, between 2005 and 2006.

The sample mostly contained state schools (*n*=7) and also included Christian schools (*n*=2), Catholic schools (*n*=2) and one independent school.^23^ Schools involved in the study were similar in size and socioeconomic status compared with the remaining 37 schools in the region.^23^ Parents or guardians provided written consent before the study, and participants were provided assent on the day of data collection. Ethics approval was granted by the Deakin University Human Research Ethics Committee (EC 37-2004). The IYM project was a registered trial (Australian New Zealand Clinical Trials Registry Number 12607000257460).

### Measures

#### Body mass index

Anthropometric data were measured by trained research staff by using a standardised procedure.^24^ Participants' heights were measured to the closest 0.1 cm by using portable stadiometers (Surgical and Medical PE87 or Leicester Height Measure, Seca Model 214, Seca, Chino, CA, USA). Weight was measured to the nearest 0.1 kg, using a TANITA body composition analyser (Model BC 418). BMI was calculated as weight (kg)/height (m^2^), and *BMI-z* was calculated using the World Health Organization (WHO) Growth Reference 2007.^25^ The WHO Reference 2007 age-specific BMI cutoffs were also used to classify children's weight status as thinness < −2 s.d., normal weight between −2 and 1 s.d., overweight between 1 and 2 s.d., and obesity >2 s.d.^25^

#### Weight perception

Weight perception was measured using one item from the Adolescent Behaviour, Attitudes and Knowledge Questionnaire, which was designed for the purpose of this study. Participants responded to the question ‘How would you describe your weight?' by choosing a response out of the following levels: 1=very underweight, 2=slightly underweight, 3=about the right weight, 4=slightly overweight or 5=very overweight. The five levels were collapsed into three: 0=about the right weight, 1=underweight and 2=overweight. The weight perception item was designed specifically for this study, and the Adolescent Behaviour, Attitudes and Knowledge questionnaire was piloted in a sample of 95 students from Australia.^24^ The pilot sample commented on the comprehensibility and readability of the questionnaire while the range of responses was checked. The survey was modified in response to this feedback.

#### Health-Related Quality of Life

HRQoL was assessed using the adolescent form of the Paediatric Quality of Life Inventory (PedsQL 4.0).^26,27^ The PedsQL 4.0 comprises 23 items, with responses assessed on a five-point Likert scale ranging from 0 (never a problem) to 4 (almost always a problem). Items are combined into a global measure of HRQoL, encompassing a physical subdomain (8 items) and a psychosocial subdomain (15 items), the latter capturing information about emotional, social and school functioning and well-being. The composite and subdomain scores range from 0 to 100, with higher scores indicating greater HRQoL. In the interest of parsimony, only global, psychosocial and physical domains were analysed in this study.

#### Covariates

Covariates were participant's gender and age. All regression models accounted for the clustering of data by school.

### Procedure

Students completed the questionnaires in class, providing demographic information via paper questionnaire and completing HRQoL with the use of Personal Digital Assistant devices (hand-held computers, Hewlett Packard iPAC Pocket PC, Hewlett Packard, Palo Alto, CA, USA).^24^ Questionnaires generally took students 30–40 min to complete. Completion of questionnaires was overseen by trained research staff who also undertook the direct measurement of anthropometric data.

### Statistical analysis

All statistical analyses were carried out using the Stata 12 (StataCorp., College Station, TX, USA) statistics package. All continuous variables were checked for normality and any responses >3 s.d. from the mean were excluded casewise from the relevant analysis. Demographic variables are presented using descriptive statistics, and gender differences were tested using *t*-tests for continuous variables and *χ*^2^ analyses for categorical variables. Some or all data were missing from 92 participants who were excluded casewise from analysis (3.03% missing data).

A series of linear regression analyses were devised to examine associations between the main effects and multiplicative interactions between the independent variables, *BMI-z* and weight perceptions, and the dependent variables and PedsQL outcome (global score, physical score and psychosocial score). Multiplicative models are consistent with the theoretical perspective of the study.^28^

An additional analysis estimated HRQoL differences, or average marginal effects (AMEs), between individuals with different weight perceptions for a given discrete *BMI z*-score between −2 and 3. This analysis generated AMEs for both overweight perception and underweight perception, compared against right weight perception for all HRQoL outcomes. Significant findings in this analysis can be interpreted as a meaningful HRQoL difference between individuals with a specified *BMI z*-score who perceive their weight as ‘about right' and individuals of the same weight status who perceived themselves to be either overweight or underweight.

The differences in the slope of *BMI-z* over the three levels of the categorical variable (weight perceptions) were tested for significance. The predicted margins for the interactions between the three-level categorical variable (weight perception) and the continuous variable (*BMI-z*) were calculated and graphed. For all tests of statistical significance, an alpha level of 0.05 was adopted.

## Results

The sample included 3040 secondary school students, surveyed during the baseline phase of IYM. Demographic characteristics along with tests of significance for gender differences are reported in Table 1. All outcome variables were found to differ by gender, except age, *BMI-z* and weight status.

### Demographic characteristics

The levels of concordance between measured weight status and self-reported weight perception are presented in Table 2, together with *χ*^2^ analysis to test the association between the two variables. Although there was a significant association between measured weight status and weight perception, there remained a clear proportion of the sample that did not accurately perceive their weight status; overall, 21.8% of participants with a BMI in the normal/thinness range thought they were underweight (<2% of the sample had a BMI in the thinness range) and 9.4% of normal weight participants thought they were overweight. The degree of distortion increased substantially for the participants with a BMI in the overweight range, where 48.7% perceived themselves as being the right weight. In contrast, almost all the participants (87.5%) who had a BMI in the obesity category correctly perceived themselves to be overweight. The patterns were largely similar among males and females, with the notable exception of females whose BMI was categorised as normal/thinness and who more often perceived themselves as overweight (13.9%) compared with males (6.0%).

There was a significant association between HRQoL and *BMI z*-score in this sample, although the strength of the association was weak by Cohen's criteria^29^ (Cohen's *r*=0.07, *P*<0.01). A significant correlation was also found between HRQoL and weight perception (*r*=0.10, *P*<0.01).

To test the hypothesis regarding interactions, we used regression analyses and AMEs analyses, and results are displayed in Table 3 and in Supplementary Tables. Supplementary Information is available at the International Journal of Obesity's website.

**Figure 1:**
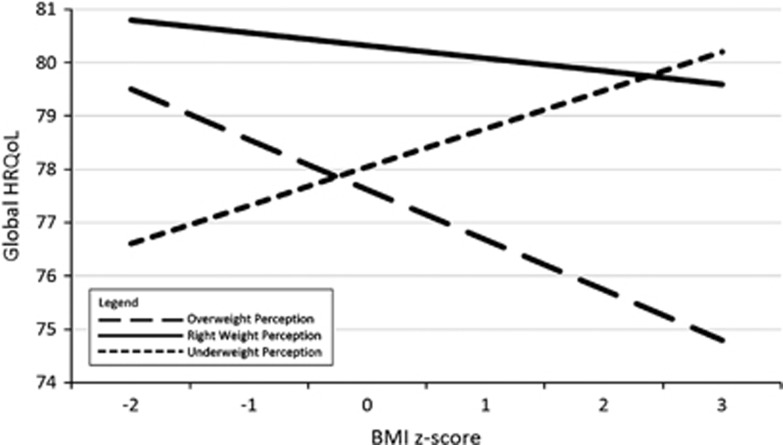
Plotted marginal effect between categorical weight perception and continuous *BMI z*-score and Global HRQoL. The figure shows the different effects of weight status on adolescents who have different perceptions of their weight. The outcome in this figure is Global HRQoL, measured in both male and female participants. The unbroken line represents adolescents who perceive themselves to be the ‘right weight,' whereas the long-dashed line represents adolescents who perceive themselves to be ‘overweight' and the short-dashed line represents adolescents who perceive themselves to be ‘underweight'.

**Figure 2:**
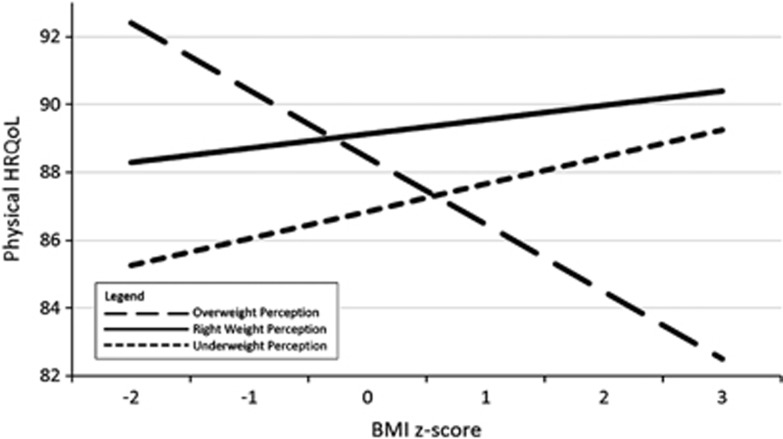
Plotted marginal effect between categorical weight perception and continuous *BMI z*-score and Physical HRQoL (male participants). The figure shows the different effects of weight status on male adolescents who have different perceptions of their weight. The outcome in this figure is Physical HRQoL. The unbroken line represents adolescents who perceive themselves to be the ‘right weight,' whereas the long-dashed line represents adolescents who perceive themselves to be ‘overweight' and the short-dashed line represents adolescents who perceive themselves to be ‘underweight'.

**Figure 3:**
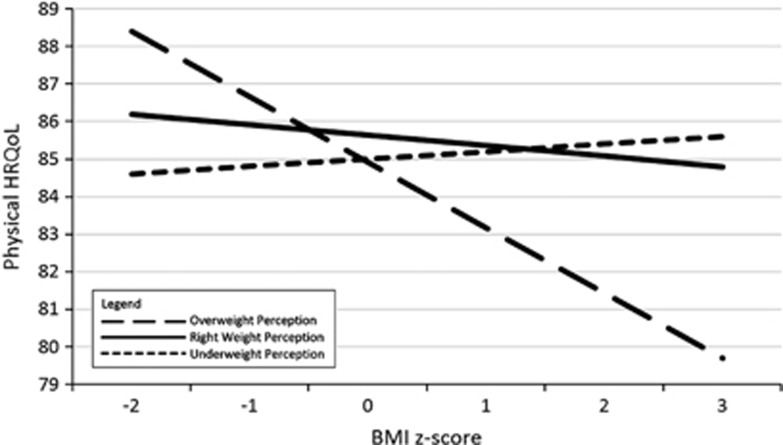
Plotted marginal effect between categorical weight perception and continuous *BMI z*-score and Physical HRQoL (female participants). The figure shows the different effects of weight status on female adolescents who have different perceptions of their weight. The outcome in this figure is Physical HRQoL. The unbroken line represents adolescents who perceive themselves to be the ‘right weight,' whereas the long-dashed line represents adolescents who perceive themselves to be ‘overweight' and the short-dashed line represents adolescents who perceive themselves to be ‘underweight'.

AME analysis (Supplementary Information available at the International Journal of Obesity's website) estimated HRQoL AMEs between right weight perceiving individuals and underweight or overweight perceiving individuals at discrete *BMI z*-scores between −2 and 3. For the purposes of further interpretation, graphs of the predicted margins for Global PedsQL score (whole sample) and Physical PedsQL score (male and female participants separately) are provided in Figures 1,2,3.

### Regression analyses

The regression analyses for Global HRQoL (Table 3) showed that there was a significant interaction between *BMI z*-score and weight perception when participants with underweight perceptions were compared with those who perceived themselves as ‘right weight' (*P*=0.04). A significant interaction was also identified in the physical subscale when participants with overweight perceptions were compared with those who perceived themselves as ‘right weight' (*P*=0.006).

When observed by gender, significant interactions were observed between females perceiving underweight vs right weight for Global HRQoL (*P*=0.02), females perceiving underweight vs right weight for Psychosocial HRQoL (*P*=0.04), and in males perceiving overweight vs right weight for Physical HRQoL (*P*=0.006).

Notably, for each significant interaction between the right weight perceivers and the relevant comparison group, the opposite comparison groups did not reach significance in the regression (that is, for Global HRQoL, *BMI z*-score × underweight vs right weight was significant, whereas *BMI z*-score × overweight vs right weight was not significant). Inspection of the regression coefficients and robust standard error shows that in these cases the effect coefficients were lower, and the error is often higher than the significant interaction terms. This suggests that the non-significant interactions may have resulted from a lack of statistical power for these comparisons.

### AME analyses—global HRQoL, underweight vs right weight perception

Examination of AMEs showed that at *BMI z*-scores in the thinness or the lower end of the normal weight range (*BMI-z*⩽0), participants who perceived themselves as underweight had lower global HRQoL than those who perceived themselves as ‘about right' (Figure 1). At *BMI z*-scores above 0, the two weight perception groups reported similar global HRQoL.

### AME analyses—global HRQoL, overweight vs right weight perception

There was a different pattern for those who perceived themselves to be overweight, when *BMI z*-scores were in the higher end of the normal range or in the overweight/obesity range (*BMI-z*⩾0), participants had lower global HRQoL than those who perceived themselves to be ‘about right' (Figure 1). There was no significant global HRQoL difference at *BMI z*-scores −2 or −1. Thus, when perceptions of overweight were concordant with measured weight, global HRQoL was lower than in participants of similar weight who perceived their weight as being ‘about right'. Examination of AMEs by gender replicated the above results in males and females separately.

### AME analyses—psychosocial functioning, underweight vs right weight perception

For psychosocial functioning, the AMEs showed that participants who reported perceptions of underweight had significantly lower scores than those who reported being about the right weight for those in the *BMI-z* range from −2 to 0 (lower end of normal weight range), but there was no significant difference from *BMI z*-scores of 0–3.

### AME analyses—psychosocial functioning, overweight vs right weight perception

Psychosocial scores for adolescents who reported overweight perceptions were significantly lower than those who reported their weight as ‘about right' at *BMI z*-scores 0–3 (high end of normal to overweight/obesity range). Estimated AMEs for these participants at *BMI z*-scores −2 and −1 (low end of normal range) were not significantly different from those who considered their weight to be ‘about right.' Therefore, when weight perception was concordant with measured weight, psychosocial scores were lower than when participants described their weight as being ‘about right.' Participants who were in the high end of the normal weight range and reported overweight perceptions also had lower psychosocial scores. When considered by gender, this pattern was consistent with both male and female participants.

### AME analyses—physical functioning

For physical scores, examination of AMEs showed that participants who described themselves as underweight recorded lower physical functioning at *BMI z*-scores −2 to 1 (lower end of normal weight range) than those who described their weight as ‘about right.' For those participants who described themselves as overweight compared with those who described themselves about the right weight, lower physical functioning scores were recorded at *BMI z*-scores 1–3 (overweight/obesity range). Again concordance between perceived weight and actual weight was associated with lower physical functioning scores.

There were differences between male and female patterns of physical functioning scores. Males who perceived themselves as underweight demonstrated significantly lower physical functioning scores at *BMI z*-scores −2 to 0 (lower end of normal weight range) compared with those who perceived themselves to be the right weight. However, females perceiving themselves as underweight showed no significant difference in physical functioning compared with those who perceived themselves as the right weight for any discrete *BMI z*-score.

These results show that adolescents who are objectively at the lower end of the *BMI z*-score spectrum and describe themselves as underweight differ according to gender. Perceptions of underweight are associated with lower physical functioning in male but not in female participants. The plotted AMEs of the interaction are presented in Figure 2 (males) and Figure 3 (females).

Effect size analysis showed that the interaction models used explained between 1.94 and 4.9% of the variance in HRQoL scores.

## Discussion

This study aimed to build upon current understanding of the relationship between adolescent obesity and HRQoL. In order to understand individual differences that might attenuate the negative association between overweight/obesity and HRQoL, the current study investigated weight perception as a potential moderator of the association between *BMI z*-score and HRQoL in adolescence, with consideration of gender differences. It was hypothesised that adolescents whose weight perceptions were concordant with an extreme objective weight status would report lower HRQoL than their peers who reported discordant weight perceptions.

To date, no studies have identified perception-related moderators of the relationship between adolescent obesity and HRQoL. The findings of this study partially supported the hypothesised moderation effect, suggesting that the association between obesity and HRQoL in adolescence significantly differs according to weight perception. Regression analyses suggested that the hypothesised interaction effect was significant only in females for global HRQoL and psychosocial functioning, and in males for physical functioning, whereas examination of AMEs supported the existence of the interaction effect in both males and females for global HRQoL and psychosocial functioning, differing by gender only for physical functioning.

Plotted estimates of the AMEs of the interaction terms revealed that, among adolescents, those who accurately perceive their objective overweight or obesity have lower global, psychosocial and physical HRQoL than those who perceive themselves as being ‘about the right weight.' Adolescents who perceived themselves as overweight and were at the higher end of the normal *BMI z*-score range (that is, *z*-scores 0–1) also appeared to have lower HRQoL than those who perceived themselves as about the right weight. Results suggested that the gap in HRQoL for these adolescents may increase as objective *BMI z*-score increases. In the lower *BMI z*-score range, there was no detectable difference between the HRQoL of adolescents who perceived themselves as ‘about right' vs overweight.

Among adolescents who had *BMI z*-scores in the lower end of the normal weight range (that is, *z*-scores −2 to 0), those who perceived themselves as underweight had lower HRQoL than those who perceived themselves as ‘about right' weight, although examination of gender differences on physical functioning revealed that this effect was evident for males only. Again, results suggest that the HRQoL divide between these groups may widen as *BMI z*-score decreases.

The findings of this study support the argument that adolescent males must not be ignored in body image and perceptions research. In this sample, the proportions of female participants who were objectively in the normal/thinness weight category, but who described themselves as either underweight or overweight, were similar (16.8 and 13.9%, respectively). By comparison, males in this category described themselves more often as underweight (25.6%) than overweight (6.0%). This finding may be explained by previous literature that found that a high proportion of adolescent males wish to increase the size of their muscles.^30^ The underweight perceptions of these students may reflect a desire to increase body weight by accumulating lean muscle mass. There is a significant body of evidence identifying points of difference between adolescent male and female body ideals;^31,32^ however, in some areas of research there remains an exclusive focus on females. This female-centric focus has fostered a gap in research that examines both male and female participants simultaneously.^33^ This study suggests that females and males are both affected by body-perception issues relating to weight. Future efforts to address adolescents' weight perception and body image problems should not focus solely on females.

In this sample, adolescents whose weight perceptions matched their objective overweight or underweight reported the lowest HRQoL. This may suggest that the effects of actual weight status on adolescent HRQoL involve social mechanisms. As adolescents become aware of their extreme weight status, HRQoL may be impacted by the internalisation of the various stigma that adolescents attribute to different or non-ideal bodies.^19,20^ This highlights the importance of adequate support networks for adolescents during this critical developmental period. Where adolescents become aware of their objective weight status, there must be support available to offset the associated reductions in HRQoL.

Two further implications for practise arose from this study. First, programs aiming to educate adolescents about healthy weight must consider the potential negative effects of altering adolescent weight perceptions. Second, obesity prevention efforts should consider whether it is appropriate to specifically target obese or at-risk adolescents, when population or community interventions may serve as a more indirect path to health promotion, without the risk of damaging adolescent HRQoL. Many of the determinants surrounding adolescent weight may be environmental, and therefore largely beyond the control of individual adolescents. Population-based programmes allow for promotion of healthy lifestyle and environmental change across the community, making behaviour change easier, and more socially desirable for at-risk individuals.^34^

Data collected in the IYM project were strong in its use of directly measured anthropometry, self-reported HRQoL measured with a valid and reliable instrument and self-reported information on weight perception. There is a well-documented tendency for individuals to under-report weight and over-report height when self-report BMI is adopted.^35^ Missing data was also minimal, with no more than 3.03% of cases missing from any of the regression models. Schools involved in the IYM project were representative of other schools in their region.

This study was limited in interpretation of the interaction effect identified. The discussions of Baron and Kenny^36^ of moderation methodology states that to maximise the interpretability of an interaction, independent variables should not be correlated; however, weight perception was weakly correlated to PedsQL outcomes in this sample. Although this does not violate the conditions for a moderation effect, it suggests that the effect may contain additional complexity beyond that considered in this analysis. Effect sizes in the analysis were also small, ranging between 1.94 and 4.9%. This effect size range may suggest that the significance of the findings is because of the large sample used. The study was further limited by the low number of participants who had objective thinness, and some non-significant results may be a reflection of this lack of thin participants. Results of analyses at this end of the weight distribution should therefore be interpreted with appropriate caution.

Data analysed in this study were cross-sectional that prevent conclusions being drawn about causality. Although there is an association between awareness of obesity and lower HRQoL, these data do not allow a conclusion to be made regarding whether awareness of obesity leads to decreased HRQoL, or low HRQoL predisposes an adolescent to be more cognisant of their true weight status. Further studies involving longitudinal cohorts should investigate these alternatives.

In the context of an increasingly overweight society, it is important to understand individual differences that may influence the degree to which adolescents with overweight and obesity experience diminished HRQoL. This study highlights the importance of weight perception as one of these differences, and highlights the possibility that obesity awareness may lead individuals to internalise negative stigma. Adequate social support during this critical developmental period may be important for improving the long-term outcomes for these adolescents.

## Figures and Tables

**Table 1:** Demographic characteristics of study participants

*Variable*	*Total* (N*=3040*)	*Male* (n*=1706*)	*Female* (n*=1334*)	P
	*M (s.d.)*	*M (s.d.)*	*M (s.d.)*	
Age	14.62 (1.38)	14.59 (1.34)	14.65 (1.42)	0.23
Height	164.84 (9.68)	167.50 (10.55)	161.44 (7.10)	**<0.001**
Weight	59.61 (13.40)	61.15 (14.33)	57.63 (11.84)	**<0.001**
BMI	21.78 (3.81)	21.59 (3.72)	22.03 (3.91)	**<0.01**
*BMI-z*	0.55 (1.04)	0.58 (1.06)	0.52 (1.03)	0.18
				
*PedsQL*				
Global	78.77 (10.40)	79.57 (10.28)	77.74 (10.47)	**<0.001**
Psychosocial	74.76 (12.33)	75.30 (12.26)	74.08 (12.38)	**<0.01**
Physical	86.38 (9.57)	87.82 (8.90)	84.56 (10.01)	**<0.001**
				
*Weight status (3-category)*	*N (%)*	*N (%)*	*N (%)*	
Thinness/normal	2026 (68.58)	1139 (68.61)	887 (68.55)	
Overweight	649 (21.97)	352 (21.20)	297 (22.95)	
Obesity	279 (9.44)	169 (10.18)	110 (8.50)	0.50
				
*Weight perception*				
Underweight	469 (15.88)	312 (18.80)	157 (12.13)	
About the right weight	1734 (58.70)	979 (58.98)	755 (58.35)	
Overweight	751 (25.42)	369 (22.23)	382 (29.52)	<**0.001**

Abbreviations: BMI, body mass index; BMI-z, standardised body mass index; M, mean; PedsQL, Paediatric Quality of Life Inventory. Bolded values are significant at *P*<0.01.

**Table 2:** Concordance between measured weight status and self-reported weight perception

*Measured weight status*	*Weight perception*			
	*Underweight*, n (%)	*Right weight*, n (%)	*Overweight*, n (%)	P
*All participants* (n=*2954*)				
Normal/thinness (*n*=2026)	441 (21.8)	1394 (68.8)	191 (9.4)	
Overweight (*n*=649)	17 (2.6)	316 (48.7)	316 (48.7)	
Obesity (*n*=279)	11 (3.9)	24 (8.6)	244 (87.5)	**<0.001**
				
*Males* (n*=1660)*				
Normal/thinness (*n*=1139)	292 (25.6)	779 (68.4)	68 (6.0)	
Overweight (*n*=352)	13 (3.7)	181 (51.4)	158 (44.9)	
Obesity (*n*=169)	7 (4.1)	19 (11.2)	143 (84.6)	**0.001**
				
*Females (n=1294)*				
Normal/thinness (*n*=887)	149 (16.8)	615 (69.3)	123 (13.9)	
Overweight (*n*=297)	4 (1.3)	135 (45.5)	158 (53.2)	
Obesity (*n*=110)	4 (3.6)	5 (4.6)	101 (92.8)	**<0.001**

Bolded values are significant at *P*<0.01.

**Table 3:** Regression analysis for *BMI z*-score × weight perception interaction

*PedsQL*	*All*			*Males*			*Females*		
	*Coef*	*Robust s.e.m.*	P	*Coef*	*Robust s.e.m.*	P	*Coef*	*Robust s.e.m.*	P
									
*Global*									
*BMI z*-score	−0.23	0.38	0.56	0.48	0.44	0.29	−1.12	0.64	0.11
									
*Weight perception (right weight*–*base)*									
Underweight	−2.25	0.47	**0.001**	−2.24	0.8	**0.018**	−1.94	0.68	**0.02**
Overweight	−2.68	1.1	**0.03**	−1.76	1.43	0.24	−3.29	1.2	**0.02**
									
*BMI z-score* × w*eight description (right weight*–*base)*									
Underweight	0.95	0.4	**0.04**	0.37	0.71	0.6	1.73	0.66	**0.02**
Overweight	−0.72	0.81	0.39	−1.7	0.98	0.11	0.39	1.15	0.74
									
*Psychosocial*									
*BMI z*-score	−0.33	0.44	0.47	0.42	0.54	0.45	−1.23	0.7	0.1
									
*Weight description (right weight*–*base)*									
Underweight	−2.58	0.52	**<0.001**	−2.41	0.87	**0.02**	−2.48	0.65	**0.003**
Overweight	−4.1	1.43	**0.01**	−2.96	1.76	0.12	−4.8	1.51	**0.009**
									
*BMI z-score* × w*eight description (right weight*–*base)*									
Underweight	1.2	0.57	0.06	0.66	0.89	0.47	1.84	0.82	**0.04**
Overweight	−0.04	0.93	0.97	−0.97	1.15	0.41	0.88	1.27	0.5
									
*Physical*									
*BMI z*-score	0.12	0.22	0.57	0.41	0.33	0.24	−0.28	0.44	0.53
									
*Weight description (right weight*–*base)*									
Underweight	−1.7	0.41	**0.002**	−2.28	0.62	**0.004**	−0.55	0.82	0.52
Overweight	−0.69	0.71	0.35	−0.6	0.97	0.55	−0.66	0.78	0.41
									
*BMI z-score* × w*eight description (right weight*–*base)*									
Underweight	0.37	0.48	0.45	0.4	0.74	0.59	0.49	0.59	0.42
Overweight	−2.04	0.59	**0.006**	−2.46	0.72	**0.006**	−1.43	0.71	0.07

Abbreviations: BMI, body mass index; BMI-z, standardised body mass index; Coef, coefficient. Bolded values are significant at *P*<0.05.
