# Supplementary material for: When ignorance is bliss: weight perception, body mass index and quality of life in adolescents
Source: Int J Obes (Lond). 2014 Jun 17;38(10):1328–34. doi: 10.1038/ijo.2014.78 (PMC4189380; doi:10.1038/ijo.2014.78)
Supplement: Supplementary Information [file ijo201478x1.doc]

Supplementary Information

Average marginal effects of weight perception by PedsQL scale

|  |  |  | All |  |  | Males |  |  | Females |  |
| --- | --- | --- | --- | --- | --- | --- | --- | --- | --- | --- |
| PedsQL Scale | BMI z | AME | 95% CI | | AME | 95% CI | | AME | 95% CI | |
| Global |  |  |  |  |  |  |  |  |  |  |
| Underweight | -2 | **-4.15** | **-5.60** | **-2.69** | **-3.00** | **-5.70** | **-0.29** | **-5.40** | **-7.88** | **-2.92** |
|  | -1 | **-3.20** | **-4.12** | **-2.28** | **-2.62** | **-4.34** | **-0.90** | **-3.67** | **-5.18** | **-2.16** |
|  | 0 | **-2.25** | **-3.17** | **-1.33** | **-2.24** | **-3.81** | **-0.67** | **-1.94** | **-3.28** | **-0.69** |
|  | 1 | -1.30 | -2.76 | 0.14 | -1.86 | -4.29 | 0.57 | -0.21 | -2.37 | 1.95 |
|  | 2 | -.361 | -2.51 | 1.79 | -1.48 | -5.12 | 2.16 | 1.52 | -1.78 | 4.82 |
|  | 3 | 0.58 | -2.31 | 3.48 | -1.10 | -6.05 | 3.84 | 3.24 | -1.28 | 7.78 |
| Overweight | -2 | -1.25 | -6.57 | 4.06 | 1.64 | -4.89 | 8.17 | -4.09 | -10.86 | 2.68 |
|  | -1 | -1.97 | -5.71 | 1.78 | -0.06 | -4.70 | 4.58 | -3.69 | -8.23 | 0.84 |
|  | 0 | **-2.68** | **-4.85** | **-.517** | -1.76 | -4.57 | 1.04 | **-3.30** | **-5.65** | **-0.95** |
|  | 1 | **-3.40** | **-4.06** | **-2.74** | **-3.47** | **-4.72** | **-2.21** | **-2.90** | **-3.74** | **-2.06** |
|  | 2 | **-4.12** | **-5.23** | **-3.01** | **-5.17** | **-6.81** | **-3.53** | **-2.50** | **-4.97** | **-0.03** |
|  | 3 | **-4.84** | **-7.50** | **-2.18** | **-6.87** | **-10.21** | **-3.52** | -2.10 | -6.77 | 2.55 |
| Psychosocial |  |  |  |  |  |  |  |  |  |  |
| Underweight | -2 | **-4.97** | **-7.24** | **-2.69** | **-3.74** | **-7.44** | **-0.04** | **-6.17** | **-9.01** | **-3.34** |
|  | -1 | **-3.77** | **-5.13** | **-2.41** | **-3.08** | **-5.36** | **-0.80** | **-4.32** | **-5.82** | **-2.83** |
|  | 0 | **-2.58** | **-3.61** | **-1.55** | **-2.42** | **-4.13** | **-0.70** | **-2.48** | **-3.76** | **-1.19** |
|  | 1 | -1.38 | -3.05 | 0.29 | -1.75 | -4.37 | 0.87 | -0.63 | -3.14 | 1.88 |
|  | 2 | -0.19 | -2.84 | 2.47 | -1.09 | -5.21 | 3.03 | 1.22 | -2.80 | 5.24 |
|  | 3 | 1.01 | -2.71 | 4.73 | -0.42 | -6.19 | 5.33 | 3.07 | -2.52 | 8.66 |
| Overweight | -2 | -4.02 | -10.44 | 2.40 | -1.02 | -8.77 | 6.72 | -6.58 | -14.33 | 1.17 |
|  | -1 | -4.06 | -8.66 | 0.54 | -1.99 | -7.55 | 3.56 | **-5.69** | **-11.00** | **-0.38** |
|  | 0 | **-4.10** | **-6.91** | **-1.30** | -2.97 | -6.41 | 0.48 | **-4.80** | **-7.76** | **-1.84** |
|  | 1 | **-4.14** | **-5.28** | **-3.00** | **-3.94** | **-5.65** | **-2.23** | **-3.91** | **-5.26** | **-2.56** |
|  | 2 | **-4.18** | **-5.39** | **-2.98** | **-4.91** | **-6.94** | **-2.88** | **-3.02** | **-5.73** | **-0.31** |
|  | 3 | **-4.22** | **-7.11** | **-1.33** | **-5.88** | **-9.80** | **-1.96** | -2.13 | -7.16 | 2.90 |
| Physical |  |  |  |  |  |  |  |  |  |  |
| Underweight | -2 | **-2.45** | **-4.52** | **-0.39** | **-3.09** | **-5.83** | **-0.35** | -1.54 | -3.98 | 0.91 |
|  | -1 | **-2.08** | **-3.33** | **-0.83** | **-2.68** | **-4.22** | **-1.14** | -1.04 | -2.76 | 0.67 |
|  | 0 | **-1.70** | **-2.51** | **-0.90** | **-2.28** | **-3.49** | **-1.07** | -0.55 | -2.17 | 1.07 |
|  | 1 | **-1.33** | **-2.56** | **-0.09** | -1.87 | -4.05 | 0.31 | -0.06 | -2.30 | 2.18 |
|  | 2 | -0.95 | -3.01 | 1.10 | -1.47 | -4.96 | 2.03 | 0.43 | -2.75 | 3.62 |
|  | 3 | -0.58 | -3.52 | 2.37 | -1.06 | -5.95 | 3.82 | 0.98 | -3.31 | 5.17 |
| Overweight | -2 | 3.39 | -0.32 | 7.10 | 4.32 | -0.29 | 8.94 | 2.19 | -2.05 | 6.44 |
|  | -1 | 1.35 | -1.20 | 3.90 | 1.86 | -1.37 | 5.09 | 0.76 | -2.10 | 3.63 |
|  | 0 | -0.69 | -2.09 | 0.71 | -0.61 | -2.51 | 1.30 | -0.66 | -2.20 | 0.87 |
|  | 1 | **-2.73** | **-3.12** | **-2.36** | **-3.07** | **-3.98** | **-2.16** | **-2.09** | **-2.75** | **-1.43** |
|  | 2 | **-4.77** | **-5.80** | **-3.73** | **-5.53** | **-6.96** | **-4.11** | **-3.52** | **-5.09** | **-1.95** |
|  | 3 | **-6.81** | **-8.98** | **-4.63** | **-8.00** | **-10.70** | **-5.30** | **-4.95** | **-7.85** | **-2.05** |
| *Note: AME = Average Marginal Effects. All comparisons made against base category: “Right weight”* | | | | | | | | | | |

This table shows the Average Marginal Effects analysis. Numbers within the table can be interpreted as the difference in HRQoL points between adolescents who perceived themselves as “the right weight”, and adolescents who perceived either underweight or overweight, at the indicated BMI z-score (identified by row). Significant differences are emboldened.
